# Early childhood development intervention and timely initiation of complementary feeding among HIV exposed infants in Tanzania: a quasi-experimental study

**DOI:** 10.3389/fnut.2026.1895144

**Published:** 2026-09-23

**Authors:** Ignas Lukanga, Amon Exavery, John Charles, Asheri Barankena, Nisela Didas, Esther Ndyetabura, Saida Salim, Tom Ventimiglia, Erica Kuhlik, Levina Kikoyo

**Affiliations:** 1 Pact Tanzania, Dar es Salaam, Tanzania; 2 Pact Inc., Washington, DC, United States

**Keywords:** breastfeeding, complementary feeding, early childhood development intervention, HIV-exposed infants, timely initiation

## Abstract

**Background:**

Timely initiation of complementary feeding for HIV-exposed infants (HEI) is essential in improving the child’s health and for ensuring the child’s nutritional requirements are met. ECD interventions have been integrated to HIV programs as one of the approaches to improve complementary feeding practices among HEI. However, there is limited evidence regarding the contribution of Early Childhood Development (ECD) intervention on timely initiation of complementary feeding among HEI in Tanzania.

**Methods:**

The study employed a post-test-only non-equivalent comparison-group quasi-experimental design. The study evaluation was conducted in 2025, with Mbeya and Tabora regions serving as the interventiongroup, and Shinyanga and Simiyu regions as the comparison group. Both project groups shared a common characteristic of being part of the Pact-led ACHIEVE project funded by the US government (2020–2026), while the intervention group received an additional ECD intervention. The study participants were 658 HEI aged 6–12 months from both the intervention and comparison groups. The outcome variable was the timely initiation of complementary feeding while The ECD intervention was the main explanatory variable of interest, and the associations were assessed using multivariable modified Poisson regression with robust standard errors.

**Results:**

The results from multivariable analysis revealed that the ECD intervention was significantly associated with a 10.9% higher prevalence of timely initiation of complementary feeding among HEI (aPR = 1.109, 95% CI: 1.033–1.191, *p* = 0.004). HEI whose male caregivers engaged in childcare had 37.6% higher prevalence of timely initiation of complementary feeding than those whose male caregivers were not (aPR = 1.376, 95% CI: 1.239–1.529, *p* < 0.001). HEI residing in urban areas had 12.0% lower prevalence of timely initiation of complementary feeding than their rural peers (aPR = 0.880, 95% CI: 0.803–964, *p* = 0.006). In addition, HEI whose caregivers had primary education had 8.0% lower prevalence of timely initiation of complementary feeding than those whose caregivers had never attended school (aPR = 0.920, 95% CI: 0.861–0.984, *p* = 0.015).

**Conclusion:**

The positive association between ECD intervention and higher prevalence ratio of timely initiation of complementary feeding among HEI may be attributed by improved caregiver nutrition knowledge and practices, male partner involvement resulted from routinely nutrition counseling sessions during paid home visits by CCWs. This finding suggests that expanding the coverage of the ECD intervention to reach more HEI, particularly those at higher risk, such as HEI living in urban areas, may help address existing barriers and contribute to improved child nutrition and overall development.

## 1 Introduction

The timely initiation of complementary feeding has been proven crucial in reducing the morbidity rate and exposure to allergic diseases among breastfeeding infants (1). According to the World Health Organization (WHO) infant feeding guideline, exclusive breastfeeding (EBF) is recommended for the first 6 months and continued breastfeeding with appropriate and responsive complementary feeding from 6 to 24 months for the general child population (1, 2). In contrast, the recommendation for breastfeeding women living with HIV differs, with guidance to cease breastfeeding at the child’s first birthday (3)

The existing body of knowledge has revealed the timing of complementary feeding to be a double-edged sword regarding the effects of both early and late introduction of complementary foods in low-resource settings. Early initiation of complementary feeding is directly associated with risks of adverse outcomes such as vertical transmission of HIV, morbidity, developmental delay, low nutritional quality of prepared mixed foods, and obesity. Late initiation is associated with food allergies, poor acceptance of new flavors, and nutritional inadequacy, as breast milk alone does not meet nutritional requirements after 6 months (4, 5).

While at the global level, efforts to promote prevention of mother-to-child transmission (PMTCT) of HIV have been successful. As the vertical transmission of HIV decreased from 23% to 11% globally. A similar trend was observed in Sub-Saharan Africa, where the vertical transmission rate has significantly declined from 27.18% to 16.90% over the past two decades (6). In line with global efforts, in 2000, Tanzania rolled out the PMTCT programme, which successfully reached 75% of pregnant women infected with HIV who were under treatment within a decade. This coverage increased to approximately 92% after two decades, with the mother-to-child transmission (MTCT) rate of HIV remaining at 11% for newborn cases (7, 8).

The noted decrease in MTCT of HIV unequivocally led to an increased number of HIV-exposed infants (HEI), reaching over one million children per 1.2 million live births by HIV-infected women annually at the global stage (9, 10). This population is at high risk of malnutrition and poor health status, especially in low-income countries. Existing research evidence indicates that mothers of HEI are at high chance of initiating complementary feeding before recommended age of 6 months compared to their HIV-negative counterparts. This is associated with the fear of vertical transmission, cultural beliefs, limited breastfeeding period, limited caregiver nutritional knowledge, and poor health-seeking behavior among parents due to stigma (11–13). For example where HEI parents and caregivers mainly rely on health facilities as the primary source of health and nutrition information, the health sector in Tanzania has an inadequate task force, with a ratio of 8.4 healthcare workers for 10,000 population, which is significantly below the WHO recommendation of 22.8 per 10,000 population, hence leading to limited access (14).

To address this challenge ECD interventions have been integrated into HIV programs in various parts of the world and demonstrating positive outcomes in overall child development (15, 16). Also in Tanzania EGPAF implemented Malezi program which integrates early stimulation interventions into maternal, newborn, and child health (MNCH) service to provide ECD services at these points to ensure that HIV-infected and exposed children attain their full growth potential (17). Aligning with the global effort on improving child development among HIV affected and HIV exposed children, Pact Tanzania with a support from Conrad N. Hilton Foundation (CNHF) implemented a 3-year ECD project (2023–2026) in Mbeya and Tabora regions of Tanzania. This was integrated into Pact’s Adolescents and Children HIV Incidence Reduction, Empowerment and Virus Elimination (ACHIEVE) project (2020–2026) in Tanzania. The intervention involved the 10 days training of community case workers (CCWs) on care for child development and integrated health and nutrition packages to support caregivers across five interrelated nurturing care framework domains; health, nutrition, responsive caregiving, early learning, and safety and security. The CCWs were further provided with developed job aids to deliver quality ECD services at family level through monthly home visits. Nutrition related services offered at household level involved counseling of caregivers on appropriate breastfeeding practices including both exclusive and complementary breastfeeding practices, meal preparation and routine nutritional status assessments and monitoring. The services provided were reported on monthly basis by CCWs through USSD system who were supervised designated ECD officers at district level to ensure appropriate and need-based ECD services are delivered at household level and quality reporting of services.

In Tanzania, about one-third of HEI are introduced with the complementary foods before recommended age of 6 months, [Ministry of Health (MoH) [Tanzania Mainland] (18)]. Despite the known risks of early or late initiation of complementary feeding among HEI such as malnutrition and morbidity, and the benefits of integrating ECD interventions within the HIV programs to improve rate of timely initiation of complementary feeding. There is paucity of findings on association between ECD intervention and timely initiation of complementary feeding in this population of interest (HEI) in Tanzania. The available studies on ECD interventions focus on assessing early stimulation practices, nutritional status, and health-related risks for a general population of children (19–22). This study intended to understand the effect of ECD interventions on the timely initiation of complementary feeding among HEI, when interventions are integrated into mainstream OVC program.

Furthermore, this study is guided by Bronfenbrenner (23) Ecological Systems Theory that comprises of five layers which explains how child development is an outcome of interconnected layers of environment systems (23). The theory explains the environmental system as a function of micro system, mesosystem, ecosystems, macro system and chrono system. These micro systems are the innermost layer which has direct contact with the child which is this context focuses on promoting enabling environment at home, positive interactions with the family members and responsive caregiving practices. The mesosystem is the second layer which is related with the secondary factors that influence child development as it involves interactions between the microsystems such as caregivers’ engagement with the service providers at community and facility levels which helps to strengthen their caregiving knowledge and improve parent to child interactions at home. Exosystem levels explain how the parents’ working environment, siblings experience and existing policies shapes child’s development outcome. While macrosystem focuses on culture, values and laws, in HIV context study plays a vital role in explaining how interventions addressed the barriers related to stigma among parents living with HIV (PLHIV) and societal perceptions on male caregivers’ engagement in child rearing practices. The outmost layer is chronosystem, which helps understanding how the major life transitions events such as marital status, family size as well as migrations to different communities influences child developmental outcome over time.

In this study the Bronfenbrenner’s ecological systems theory shows the linkage between Pacts’ ECD intervention and timely initiation of complementary feeding at different levels by creating enabling environment at family and community level. Similarly the study by Nîmante (24) affirms that childhood developmental outcome is not only determined by genetic make-up but external factors such as family, community and broader environmental context.

## 2 Material and methods

### 2.1 Study design and data source

This study is part of an ECD Outcomes Evaluation study, which employed a post-test-only non-equivalent comparison-group quasi-experimental design, in which two groups were compared: Mbeya and Tabora regions as the ECD intervention group, and Shinyanga and Simiyu regions as the non-ECD comparison group. Both groups shared the common characteristic of being part of the Pact-led Adolescents and Children HIV Incidence Reduction, Empowerment and Virus Elimination (ACHIEVE) project funded by the US government, and implemented in 16 regions in Tanzania from year 2020 to 2026. The ACHIEVE project focuses primarily on improving HIV-related outcomes among four priority subpopulations which includes HIV exposed infants (HEI), children living with HIV (CLHIV), pregnant and breastfeeding women (PBFW) and parents living with HIV (PLHIV). ACHIEVE project uses a comprehensive, family-centered case management approach led by community-based case workers to provide a broad package of services to households with orphans and other vulnerable children in close coordination with the government of Tanzania’s health and social services and other service providers.

The intervention group in this study was the one that received additional Early Childhood Development (ECD) services under an integrated activity funded by the Conrad N. Hilton Foundation, 2023–2026. The ECD intervention aimed to increase access to quality ECD services for the households of Orphans and Vulnerable Children (OVC) aged 0–3 years. By integrating ECD services into the case management approach, the project ensured that families received comprehensive support across the five domains of the Nurturing Care Framework: health, nutrition, safety and security, opportunities for early learning, and responsive caregiving, which help children reach their full potential. Except for the additional ECD services provided to the intervention region, the selected study sample across the two groups had similar background characteristics, such as participation in the ACHIEVE OVC program, being born to an HIV-positive mother, and being in the age category of 6–12 months, as well as cultural and social alignments.

### 2.2 Study area

The study was conducted across four regions of Tanzania, namely Mbeya and Tabora regions representing the intervention regions, and Shinyanga and Simiyu regions representing the comparison regions. All four regions were part of 16 regions of ACHIEVE project implementation, while the former were purposely selected because they received additional ECD interventions through the ECD project (supported by Conrad N. Hilton Foundation) integrated into the ACHIEVE OVC program (funded by the US government). The latter were selected for their similarity to the intervention regions in key characteristics, such as being recipients of the ACHIEVE OVC program, HIV/AIDS prevalence, demographic characteristics, and similar vulnerability profiles.

### 2.3 Study participants

The study participants comprised HEI aged 6–12 months. A HEI in this case referred to a child born to or breastfed by a woman living with HIV. HEI from the intervention group participated in the ACHIEVE and ECD projects, while those in the comparison group participated in the ACHIEVE project only. On the other hand, all OVC who were aged below 6 months or above 12 months were excluded because they were at the age of exclusive breastfeeding and weaning, respectively. In addition, those with missing complementary feeding data were excluded from the analysis. These inclusion and exclusion criteria were based on WHO recommendations regarding the appropriate age for complementary feeding of HEI (1).

### 2.4 Sampling procedures and sample size

As presented in Figure 1, the sample for this study was derived from the large sample of the ECD outcomes evaluation, where the study participants were determined through random sampling techniques. To ensure comparability between the two groups, a 1:1 intervention-to-comparison ratio was applied. This yielded a random sample size of 1,039 households per group, resulting in a total of 2,078 households with a total of 2,277 children aged 0–3 years across the intervention and comparison regions. After applying the inclusion and exclusion criteria, the final sample size for the present study remained 658, where 357 HEI represented the comparison group (i.e., HEI who benefited from ACHIEVE project services only) and the other 301 represented the intervention group (i.e., HEI who received both ACHIEVE and ECD intervention services).

**FIGURE 1:**
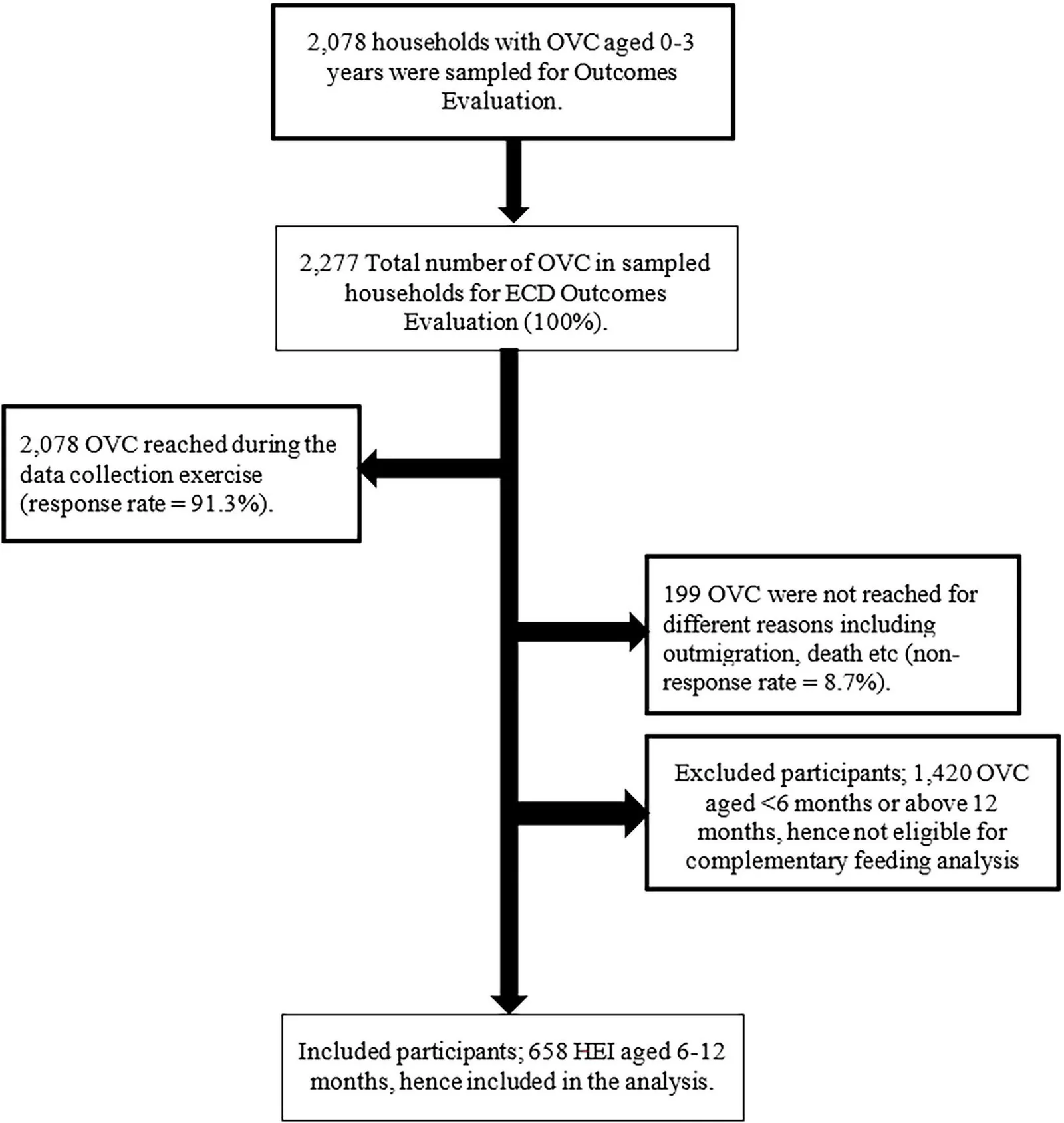
A sampling procedure.

### 2.5 Variables

The response variable in this study was a dummy variable representing timely initiation of complementary feeding, coded “1” for HEI who were initiated with complementary feeding on time, and “0” for those who were not initiated timely. An infant is considered to have been timely initiated with complementary feeding if it was initiated between the ages of 6–8 months (3). On the other hand, if an infant was initiated complementary feeding before the age of 6 months (based on their retrospective records) and after 8 months, it was considered not timely initiated complementary feeding, Equation (1).


Initiation⁢of⁢complementary⁢feeding= (1)

{ 0,if⁢not⁢timely⁢initiated⁢(before⁢ 6⁢or⁢after⁢ 8⁢months⁢of⁢age)1,if⁢timely⁢initiated⁢(within⁢ 6−8⁢months⁢age⁢period)

The primary independent variable was the ECD intervention status, where Mbeya and Tabora regions constituted the intervention group, and Shinyanga and Simiyu regions served as the comparison group. Several other independent variables were included in the analysis as potential confounders. These were HEI age, HEI sex, caregiver HIV status, caregiver age, caregiver sex, caregiver marital status, caregiver education, CCW sex, household family size, and place of residence.

### 2.6 Data collection

The data collection for this was done by CCWs through face-to-face interviews using a structured questionnaire, along with observations and routine program data. For this study, the ECD monthly service tracking form and ECD enrollment form were used. Data for timely initiation of complementary feeding was drawn from ECD project’s monthly service tracking form and on the other end ECD Enrollment form captured the age at which OVC was enrolled in ECD project and other demographic information, to help tracking of age-appropriate ECD services and reduce bias in the USSD reporting.

### 2.7 Data analysis

Data analysis for this study began with descriptive statistics, including the frequency distribution of key variables to summarize the study population. Bivariate analysis was then conducted to examine differences in timely complementary feeding by ECD intervention status and other background characteristics using a Chi-square (χ^2^) test. Following this, multivariable analysis was performed using modified Poisson regression with robust standard errors, with timely complementary feeding as the dependent variable. This approach was selected because the outcome (i.e., timely initiation of complementary feeding) was common, allowing adjusted prevalence ratios (aPRs), with corresponding 95% confidence intervals and *p*-values, to be estimated directly. Clustering at the household level was accounted for among children residing in the same household, while there was no evidence of clustering at higher levels. ECD intervention status served as the primary independent variable, while additional covariates were included in the model as potential confounders. All analyses were conducted using Stata version 18.0. Statistical significance was set at 5%, and variables with *p*-values of 0.05 or less were considered statistically significant.

## 3 Results

### 3.1 Characteristics of respondents

As presented in Table 1, the study included a total of 658 HEI aged 6–12 months, with 45.74% and 54.26% from intervention and comparison regions, respectively. Of these, 498 (75.68%) were from rural settings and 160 (24.32%) from urban areas. Results in Table 1 indicated that the majority of HEI (70.82%) had their male caregivers participate in childcare practices. Additionally, the sample of HEI was evenly distributed with regard to sex, with 50.61% female and 49.39% male. The majority of children came from smaller family sizes of 2–3 people (52.42%), followed by a medium family size of 4–6 people (43.16%), then a larger family size of 7+ people (4.40%). A greater proportion of HEI 57.91% were served by female CCWs; a similar trend was observed among caregivers, with 97.59% being female.

**TABLE 1:** Profile of respondents (*n* = 658).

Background characteristics	*n* (%)
Overall	658 (100.0%)
ECD intervention status	
Comparison group	357 (54.26%)
Intervention group	301 (45.74%)
Initiation of complementary feeding	
Not timely	111 (16.87%)
Timely	547 (83.13%)
Male involvement in childcare	
No	192 (29.18%)
Yes	466 (70.82%)
Residence	
Urban	160 (24.32%)
Rural	498 (75.68%)
Family size	
2–3 people)	345 (52.42%)
4–6 people	284 (43.16%)
7+ people	29 (4.40%)
HEI sex	
Female	333 (50.61%)
Male	325 (49.39%)
Caregiver age	
<30 years	186 (28.26%)
30–39 years	339 (51.51%)
40–49 years	120 (18.24%)
50+ years	13 (1.98%)
CCW Sex	
Female	381 (57.91%)
Male	277 (42.09%)
Caregiver HIV status	
Positive	602 (91.49%)
Negative	56 (8.51%)
Caregiver sex	
Female	642 (97.59%)
Male	16 (2.41%)
Caregiver education	
Never attended	77 (11.7%)
Primary education	557 (84.65%)
Secondary education	24 (3.65%)

### 3.2 Complementary feeding by background characteristics

Table 2 presents the results from the bivariate analysis examining the association between the timely initiation of complementary feeding and its background characteristics. Overall, 83.13% of HEI were timely initiated on complementary feeding. The study found a significant variation in the rate of timely initiation of complementary feeding across the examined background characteristics. For instance, the proportion of timely initiation was significantly higher in the intervention group (87.38%) compared to the comparison group (79.55%) (*p* = 0.008). Timely initiation of complementary feeding also varied across several background characteristics, including male involvement in childcare (*p* = 0.001), residence (*p* = 0.008), and caregiver education (*p* = 0.023).

**TABLE 2:** Percentage of HEI with and without timely complementary feeding by ECD intervention status by background characteristics (*n* = 658).

	Complementary feeding initiation		Chi-square	*P*-value
Background characteristics	Not timely initiated	Timely initiated		
	(*n* = 111)	(*n* = 547)		
Overall	111 (16.87%)	547 (83.13%)		
ECD intervention status				
Comparison regions	73 (20.45%)	284 (79.55%)	7.1279	0.008
Intervention regions	38 (12.62%)	263 (87.38%)		
Male involvement in childcare				
No	66 (34.38%)	126 (65.62%)	59.2436	<0.001
Yes	45 (9.66%)	421 (90.34%)		
Residence				
Urban	38 (23.75%)	122 (76.25%)	7.1371	0.008
Rural	73 (14.66%)	425 (85%)		
Family size				
2–3 people	61 (17.68%)	284 (82.32%)	2.2191	0.33
4–6 people	48 (16.9%)	236 (83.10%)		
7+ people	2 (6.90%)	27 (93.10%)		
HEI sex				
Female	60 (18.02%)	273 (81.98%)	0.6344	0.426
Male	51 (15.69%)	274 (84.31%)		
Caregiver age				
<30 years	29 (15.59%)	157 (84.41%)	0.3402	0.952
30–39 years	59 (17.40)	280 (82.6%)		
40–49 years	21 (17.50%)	99 (82.5%)		
50+ years	2 (15.38%)	11 (84.62%)		
CCW sex				
Female	62 (16.27%)	319 (83.73%)	0.2295	0.632
Male	49 (17.69%)	228 (82.31%)		
Caregiver HIV status				
Positive	104 (17%)	498 (82.72%)	0.8333	0.361
Negative	7 (12.50%)	49 (87.50%)		
Caregiver sex				
Female	108 (16.82%)	534 (83.18%)	0.0414	0.839
Male	3 (18.75%)	13 (81.25%)		
Caregiver education				
Never attended	5 (6.5%)	72 (93.5%)	7.5093	0.023
Primary education	100 (17.95%)	457 (82.05%)		
Secondary education	6 (25%)	18 (75%)		

### 3.3 Results from multivariable analysis

The results from the multivariable analysis (Figure 2) indicated that after adjusting for several factors, children in the ECD intervention regions had 10.9% higher prevalence of timely initiation of complementary feeding than those in the comparison regions (aPR = 1.109, 95% CI: 1.033–1.191, *p* = 0.004) (Figure 2). On the other hand, HEI whose male caregivers engaged in childcare had 37.6% higher prevalence of timely initiation of complementary feeding than those whose male caregivers did not engage in childcare (aPR = 1.376, 95% CI: 1.239–1.529, *p* < 0.001). Additionally, the study found that HEI residing in urban areas had 12.0% lower prevalence of timely initiation of complementary feeding than their rural peers (aPR = 0.880, 95% CI: 0.803-0.964, *p* = 0.006). In addition, HEI whose caregivers had primary education had 8.0% lower prevalence of timely initiation of complementary feeding than those whose caregivers had never attended school (aPR = 0.920, 95% CI: 0.861–0.984, *p* = 0.015).

**FIGURE 2:**
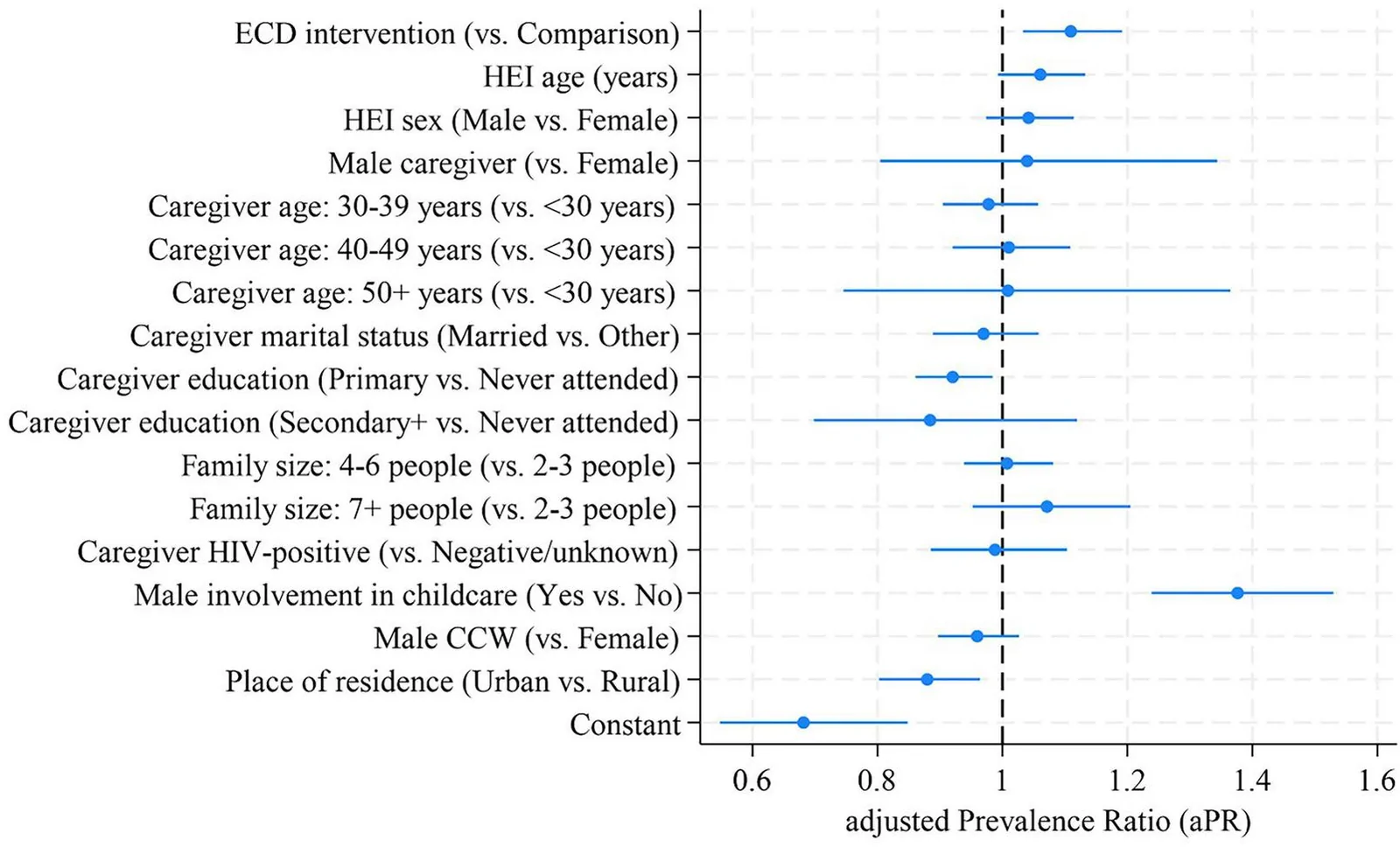
Multivariable modified Poisson regression analysis of factors associated with timely initiation of complementary feeding among HIV-exposed infants aged 6–12 months in Tanzania (*n* = 658).

## 4 Discussion

The study assessed the association between participation of HEI households in an ECD intervention and the timely initiation of complementary feeding among HEI by comparing the ECD intervention and non-ECD intervention regions. It was found that participation in the ECD intervention was significantly associated with the timely initiation of complementary feeding, with 87.38% of HEI in sampled households from the intervention region being initiated on time, compared to 79.55% of HEI in the comparison region. Additionally, the study revealed that the prevalence of timely initiation of complementary feeding among HEI was significantly higher in intervention regions than in comparison regions. The current rates of timely initiation of complementary feeding among HEI and the general infant population in Tanzania stand at 41% and 89%, respectively [(25); (Ministry of Health (MoH) [Tanzania Mainland] (18)]. The observed performance in the intervention regions underscores that the integrated ECD services may have helped in addressing existing challenges, such as stigma and poor health-seeking behavior, as well as in strengthening caregivers’ nutrition knowledge. These improvements resulted in rates of complementary feeding that were nearly comparable to those of the general infant population. Similar findings were reported by Haque et al. (26) showing that those households enrolled in ECD interventions delivered through home visiting experienced significantly improved timely initiation of complementary feeding compared with their non-intervention counterpart.

The study found a significant association between male caregivers involved in early childhood development activities and the timely initiation of complementary feeding among HEI. In most areas, male caregivers involvement in complementary feeding is reported to be low and even lower within the context of HIV (20, 27). The observed positive outcome on timely initiation of complementary feeding as a result of male involvement in childcare could be due to effectively addressing the existing cultural barriers and perceptions that such responsibilities are solely for female caregivers (28). It could also result from a reduction of household chores needed to be done by mothers and offering them more time to focus on child rearing, as well as joint decision-making on food choices (29). Studies from elsewhere have indicated that when male caregivers are empowered through ECD interventions, they substantially influence the timely initiation of complementary feeding (30). Similar findings were reported by Etowa et al. (31), who found that male partners’ involvement in child-rearing within the HIV context reduces the risks of mixed feeding and encourages positive feeding outcomes among infants.

This study reveals that HEI residing in urban areas had a significantly lower prevalence of timely initiation of complementary feeding than their rural counterparts. Additionally, the proportion of timely initiation of complementary feeding among HEI residing in rural and urban settings was 85% and 76%, respectively. The higher proportion of timely initiation in rural settings aligns closely with the 86.8% reported by the Tanzania National Nutrition Survey [Ministry of Health, Community Development, Gender, Elderly and Children (MoHCDGEC) [Tanzania Mainland] (32)]. Similar findings were also reported by Masuke et al. (33) where much lower compliance with the timely initiation of complementary feeding was observed in Moshi Urban, northern Tanzania, with 91% of children being introduced to semi-solid foods before the age of 6 months. Additionally, the study by Sichalwe et al. (34) in Kinondoni municipal, coastal zone of Tanzania, found that only 43% of mothers timely initiated complementary food to their children. Even though Pact’s ECD intervention provided similar services for both rural and urban areas, using the same mode of delivery, the variation could be attributed to the advantage of common socio-economic activities, such as farming, and cultural uniformity in rural settings, which facilitate the organization of and encourage participation in ECD interventions and thus enhance knowledge-sharing. Findings from other similar studies also suggest that the cultural and socio-economic regularities in rural areas aid the seamless implementation of ECD interventions (35, 36). Conversely, urban settings face disadvantages due to busy work schedules, cultural diversity, and fears of HIV status disclosure, which may limit effective participation in ECD interventions and overall complementary feeding practices.

The current study observed that HEI whose caregivers have never attended school had higher prevalence of timely initiation of complementary feeding than those whose caregivers had primary education or more. This finding contrasts with the expectation that education increases caregivers’ awareness and exposure, thereby improving overall feeding practices. The findings in the current study conflict with those reported by Celebier et al. (37), who found that families with a lower educational background did not timely initiate complementary feeding as recommended. Similarly, the study by (38) reported a high likelihood of prelacteal feeding among infants of illiterate fathers. The unexpected association observed in this study is still unclear as to whether the caregivers with formal education are attached to some employment that limits their breastfeeding time. Hence, this area can be further researched to determine the associated reasons and address the barriers.

## 5 Strengths and limitations

Although this study was based on a robust design (quasi-experimental) and employed rigorous statistical analyses, its findings are limited by the inclusion of only four regions, which restricts the generalizability of the results beyond the study area. Also, given the non-randomized study design, causal inferences cannot be established beyond the observed associations. Additionally, residual confounding remains possible, particularly due to factors that were not available for inclusion in the analysis, such as socioeconomic status.

## 6 Conclusion

The study found that the prevalence of timely initiation of complementary feeding among HEI was significantly higher among HEI in the intervention group than among those in the comparison group. A similarly higher prevalence was observed among HEI whose male caregivers engaged in childcare. However, the prevalence of timely initiation of complementary feeding was lower among HEI residing in urban areas and those whose parents had primary or secondary education. These findings suggest that expanding the coverage of the ECD intervention to reach more HEI, particularly those at higher risk, such as HEI living in urban areas, may help address existing barriers and contribute to improved child nutrition and overall development.

## Data Availability

The datasets presented in this article are not readily available because they involve orphaned and vulnerable children and their caregivers and are therefore unsuitable for public sharing. Requests to access the datasets should be directed to AB, abarankena@pactworld.org.
